# Nutrient Intakes of Children: Associations with Maternal Use of Pressure to Eat and a Healthy Home Food Environment

**DOI:** 10.3390/nu13062082

**Published:** 2021-06-18

**Authors:** Hebah Alawi Kutbi

**Affiliations:** Clinical Nutrition Department, Faculty of Applied Medical Sciences, King Abdulaziz University, P.O. Box 80215, Jeddah 21589, Saudi Arabia; hkutbi@kau.edu.sa

**Keywords:** feeding practices, children, pressure to eat, food environment, nutrient intake, Saudi Arabia

## Abstract

Maternal feeding practices have been shown to have a powerful influence on shaping dietary behaviors of children. Nonetheless, the association with the nutrient intake of children has not been previously explored. This study aimed to investigate the associations of maternal use of pressure to eat (PE) and a healthy home food environment (HHFE) with nutrient intakes in children (6–12 years old). Data of 424 mother–child dyads were included. Maternal use of PE and a HHFE were assessed using a validated questionnaire. Child food intake was collected using telephone-administered 24 h recalls. Multivariate linear regression analyses were conducted to investigate the associations of maternal feeding practices with energy and nutrient intakes of children. Maternal use of PE predicted higher children’s intake of carbohydrate (B = 0.90, 95% confidence interval (CI) 0.19 to 1.62) and dietary fiber (B = 1.25, 95% CI 0.32 to 2.18) and lower fat intake (B = −0.74, 95% CI −1.37 to −0.11). Maternal use of a HHFE was significantly positively associated with protein intake (B = 0.99, 95% CI 0.53 to 1.46) and negatively associated with free sugar intake in children (B = −3.56, 95% CI −5.03 to −2.09). Our findings indicate that nutrient intakes of children are linked to feeding practices employed by mothers. Further studies are warranted to gain a more contextualized understanding of mother–child feeding interactions and to develop effective interventions.

## 1. Introduction

A healthful balanced diet during childhood is essential to support growth and development, prevent chronic diseases, and maintain healthy weight status [1]. However, recent data indicate that most children in Saudi Arabia fail to comply with dietary guidelines [2]. Even though multiple factors have been suggested to play a role in child diet quality, the current evidence points to the parental role as being a key influence [3,4].

Parental feeding practices, specifically pressure to eat (PE) and the promotion of a healthy food environment, have been linked to the food intake and dietary behaviors of children [3,5,6,7]. In fact, abundant research has examined the role of maternal feeding practices in shaping dietary behaviors of children. Nonetheless, limited data exist on the relationship between maternal feeding and children’s nutrient intake. Findings from longitudinal and laboratory-based studies suggest that children exposed to a high level of PE are more likely to consume calorie-dense foods and sugar-sweetened beverages [7]. Studies have also found that a child who is exposed to greater levels of PE is less likely to eat in the absence of hunger and more likely to exhibit a picky eating behavior [8,9].

A healthy food environment can promote healthy eating behaviors. For instance, greater accessibility and availability of fruits and vegetables at home were found to be associated with higher consumption by children in many studies [5,6]. Providing a healthy home food environment (HHFE) has also been linked to improved diet quality in children [3]. In contrast, access to unhealthy food and snacks was found to be correlated with higher energy intake [10]. To develop effective clinical and community-based interventions aiming to improve the dietary intake of children, it is important to understand and consider the parent–child feeding dynamic and its association with child nutrient intake.

In order to maintain healthy nutritional status and to support normal growth and development, children must meet nutrient recommendations, which could be achieved through the consumption of healthy balanced diets consisting of a variety of foods [11]. During childhood, parents are considered responsible for what the child eats [12]. In the Middle East, mothers are often considered the primary parent responsible for feeding the child [13]. Thus, given the established associations between maternal feeding practices and dietary behaviors of children, we sought to investigate the associations of maternal use of PE and a HHFE with the nutrient intakes of children.

## 2. Materials and Methods

### 2.1. Study Sample

We intended to recruit a minimum of 346 children and their mothers. The required sample size was calculated based on a power of 80%, alpha = 0.05 (two-sided), and an expected correlation coefficient between children’s energy intake and average score of maternal feeding practices of 0.15 [14]. An online questionnaire was circulated through social media channels accompanied by an invitation for mothers of children aged 6 to 12 years old to participate in the study. The objectives of the study were listed along with the study protocol, consent for participation, sociodemographic data, questions on maternal feeding practices, and suitable date and time for communication. Dyads were eligible if the child was a Saudi citizen, residing in Saudi Arabia, medically healthy, and aged between 6 and 12 years. Children with a food allergy, autism, Down syndrome, or a chronic disease were excluded. The final analysis included data from 424 mother–child dyads. The following phase was to conduct phone interview(s) to collect 24 h dietary recall(s). Ethical approval to conduct the study was issued by the Research and Ethics Committee of the Faculty of Applied Medical Science at King Abdulaziz University (FAMS-EC-2020-0010).

### 2.2. Sociodemographic and Child Variables

Mothers provided information regarding region of residence, child’s age and sex, order of the child among siblings, maternal employment status, age, weight and height, monthly household income, maternal and paternal education status, breastfeeding initiation during the first six months of the child’s life, and whether the father resides in the household with the child and is involved in child feeding. The body mass index (BMI) of mothers was later calculated as weight (kg)/height (meter)^2^ [15].

### 2.3. Study Outcomes: Dietary Intakes of Children

Dietary data of children were collected using telephone-administered 24 h dietary recall. A subsample of 168 children (39.6%) were requested to report two weekdays and one weekend day dietary recalls to adjust for within-individual variation. A text message was sent to each mother one day prior to the scheduled phone interview as a reminder and to reschedule the interview if needed. At the telephone interview, mothers were educated on the information needed for the dietary recall. For instance, pictures of serving tools were sent to the mothers to explain how to appropriately report the portion size of the food consumed. They were also instructed to provide the type of food, such as whole versus low-fat milk, and name of manufacturer of prepackaged foods. They were also requested to have the child and people who assist in child feeding nearby to participate in the interview. Mothers were later contacted to collect the past 24 h dietary recalls.

Dietary data were entered and analyzed using Nutritics software, version 5.09 (Dublin, Ireland), which includes a database for popular Arabic foods and recipes. If a local recipe was not available, standardized recipes were manually entered by trained dietetic professionals. Nutrient densities were later calculated and reported; macronutrients and free sugar were expressed as proportions of energy intake, whereas micronutrients were expressed as the intake in unit per 1000 kcals.

### 2.4. Primary Predictors: Maternal Feeding Practices

Maternal use of PE and provision of a HHFE were evaluated using subscale items of the validated Comprehensive Feeding Practices Questionnaire (CFPQ) [13,16]. Both scale items were shown to have good internal consistency among our sample (Cronbach’s α = 0.70).

The extent to which mothers were using PE was evaluated using four statements: “My child should always eat all of the food on his/her plate”; “If my child says, ‘I’m not hungry’, I try to get him/her to eat anyway”; “If my child eats only a small amount of food, I try to get him/her to eat more”; and “When he/she says he/she is finished eating, I try to get my child to eat one more (two more, etc.) bite of food.” Maternal use of a HHFE was assessed using the following statements: “Most of the food I keep in the house is healthy”; “I keep a lot of snacks (potato chips, Doritos, cheese puffs) in my house (reverse coded)”; “A variety of healthy foods are available to my child at each meal served at home”; and “I keep a lot of sweets (candies, ice cream, cake, pies, pastries) in my house (reverse-coded)”. All items were rated by mothers on a 5-point scale, ranging from “strongly disagree” to “strongly agree”, and the average scale scores indicated the extent to which mothers are utilizing the feeding practice.

### 2.5. Statistical Analysis

Statistics were described as frequency (%) and mean ± standard deviation. Mann–Whitney and Kruskal–Wallis tests were used to evaluate the univariate associations of maternal feeding practices with categorical variables (2-sided tests). Spearman’s correlation coefficient (r_s_) was used to evaluate the correlations between energy and nutrient intakes of children and maternal feeding practices.

Multivariate linear regression analyses were performed to evaluate the associations of energy and nutrient intakes of children with (1) maternal use of PE (covariates included child age and sex, maternal age, and region of residence) and (2) maternal use of a healthy food environment (covariates included child age and sex, region of residence, order of child, paternal education, paternal involvement in feeding, father residence in child’s home, and breastfeeding status during first six months of child’s life). The analyses were carried out at a 95% confidence level using SPSS (IBM Corp., Armonk, NY, USA).

## 3. Results

### 3.1. Maternal and Child Characteristics

The child characteristics and the associations with maternal feeding practices are presented in Table 1. The sample included 144 children (34.0%) aged between 6 and 7 years, 126 children (29.7%) aged between 8 and 9 years, and 154 children (36.3%) aged 10 and 12 years. The mean age of the children was 8.67 ± 1.85 years old, of whom 49.5% were boys (*n* = 210). Approximately two thirds of fathers (63.2%, *n* = 268) and three quarters of the mothers (75.3%, *n* = 319) had completed a college degree or higher. Monthly household income for 54.0% of the children (*n* = 195) was >10,000 Saudi Riyal. Maternal age ranged between 23 and 68 years, with a mean of 37.2 ± 6.58 years. The mean maternal BMI was 26.6 ± 4.92 kg/m^2^. The majority of mothers (87.0%) reported that their children were breastfed to some extent during the first six months of their lives (*n* = 369). The mean scale scores of PE and HHFE feeding practices were 2.74 ± 1.01 and 3.45 ± 0.84 out of a total score of 5, respectively. No correlation was observed between maternal feeding practices (r_s_ = −0.02, *p* = 0.65).

The univariate analyses indicated significant negative correlations between the PE scale score and child and maternal age (r_s_ = −0.20 and r_s_ = −0.14, *p* < 0.01). The mean scale scores of the HHFE among middle siblings (3.55 ± 0.87), first siblings (3.50 ± 0.84), youngest siblings (3.35 ± 0.77), and only children (3.15 ± 0.90) were found to be significantly different (*p* = 0.04). Significant differences in healthy food environment scale scores were also observed among children whose fathers never participate in feeding (3.22 ± 0.82), sometimes participate in feeding (3.38 ± 0.80), and always participate in feeding the child (3.61 ± 0.84), *p* < 0.01. A higher paternal education level was associated with greater maternal use of a HHFE, wherein the mean scale scores of a HHFE in children of fathers who had less than a high school, high school, college, and post-graduate degree were 3.35 ± 0.92, 3.37 ± 0.83, 3.53 ± 0.84, and 3.62 ± 0.77, respectively (*p* = 0.01). Furthermore, the mean scale score of a HHFE was significantly higher when fathers always live with children (3.48 ± 0.85) compared to when fathers sometimes (3.41 ± 0.69) or never (3.07 ± 0.70) live with their children, *p* = 0.03. Additionally, the mean of the healthy food environment scale score in breastfed children (3.49 ± 0.84) was significantly higher than that of children who were never breastfed (3.22 ± 0.82) during the first six months of their lives (*p* = 0.02).

### 3.2. Associations of Maternal Feeding Practices with Dietary Intakes of Children

Mean energy intake of children in our sample was 1312 ± 348 kcals. The correlations of energy and nutrient intake with maternal feeding practices are illustrated in Table 2. The PE scale score was positively correlated with carbohydrate (r_s_ = 0.12, *p* = 0.02), fiber (r_s_ = 0.13, *p* = 0.01), total sugar (r_s_ = 0.11, *p* = 0.02), iron (r_s_ = 0.10, *p* = 0.04), and vitamin C (r_s_ = 0.11, *p* = 0.02) intakes, whereas a negative correlation with fat intake was observed (r_s_ = −0.17, *p* = 0.03). The HHFE scale score was correlated positively with protein intake (r_s_ = 0.19, *p* < 0.01) and negatively with fat (r_s_ = −0.10, *p* = 0.04), total sugar (r_s_ = −0.13, *p* = 0.01), and added sugar consumption (r_s_ = −0.27, *p* < 0.01).

Multivariate linear regression analyses were conducted to investigate the independent associations of maternal feeding practices with nutrient intakes of children (Table 3). The PE predicted a greater consumption of carbohydrate (B = 0.90 (95% CI 0.19 to 1.62)) and dietary fiber (B = 1.25 (95% CI 0.32 to 2.18)) in children and lower fat intake (B = −0.74 (95% CI −1.37 to −0.11)). Providing a HHFE was found to be significantly positively associated with protein intake in children (B = 0.99 (95% CI 0.53 to 1.46)) and negatively associated with free sugar intake (B = −3.56 (95% CI −5.03 to −2.09)), Table 4.

## 4. Discussion

Maternal use of PE and offering a HHFE have been reported to be associated with eating behaviors of children in Saudi Arabia [17]. However, the relationships with the dietary intakes of Saudi children have not been previously explored. Our data indicated that PE as a feeding practice predicts higher consumption of carbohydrate and dietary fiber and lower fat intake among children, whereas providing a HHFE was found to be associated with higher protein intake and less free sugar consumption.

Child eating behaviors are mostly shaped at home; thus, the potential impacts of the home food environment and maternal feeding practices on child nutrient intakes are particularly relevant [10,12,18]. A previous study examined the association between healthy food availability at home and diet quality of children, wherein the diet quality was calculated according to the mean daily servings consumed from eight food groups; greater availability of healthy food at home was found to be associated with a greater diet quality score [3]. In the present study, providing a HHFE was found to be associated with higher consumption of protein and less consumption of free sugar, suggesting a potentially favorable impact on a child’s dietary behaviors. On the other hand, despite the existing evidence that points at pressure feeding as a counterproductive practice on dietary behaviors of children [19], the associations observed between maternal use of PE and fiber and fat intakes in children suggest that maternal pressure in feeding might be useful in promoting the intake of certain nutrients.

Research exploring the relationship between parental use of PE eat and children’s dietary intake has revealed inconclusive results. For example, findings from laboratory-based studies demonstrated that exposing children to higher levels of PE is associated with a lower likelihood of eating healthy foods and high-fat foods [20,21]. A cross-sectional mother–daughter dyads study also explored the role of maternal use of PE on daughters’ food intake; daughters who were exposed to greater levels of PE exhibited lower consumption of fruits and vegetables and higher consumption of fat than those exposed to lower levels [18]. Contrary to these findings, some studies have shown that PE is positively associated with fruit and vegetable consumption [19,22,23], while others reported non-significant relationships [24]. In the present study, maternal PE was positively associated with carbohydrate and fiber intakes and negatively associated with fat intake. A recent study conducted in Saudi Arabia reported inadequate intake of dietary fiber and high consumption of fat among children [2]. Given that no association was observed between maternal use of PE and free sugar intake, it is speculated that a higher carbohydrate intake represents a higher consumption of starch, such as rice, bread, potato, pasta, fruit and vegetables, etc., rather than high-sugar foods and drinks. Although the findings of our study show some plausible associations between PE in childhood and fiber and fat intakes, it remains unclear whether children would adopt these eating behaviors, or they would avoid the food they were pressured to eat or alter these eating behaviors when they have a choice [9].

Many studies have focused on the maternal role in child feeding given that mothers are often recognized as the parent responsible for child feeding, particularly in the Arab countries [13,17,25,26]. Unexpectedly, our data indicated that approximately half of the fathers participate in child feeding, and paternal education level, residency at child home, and involvement in feeding were associated with greater maternal use of a HHFE. These finding shed light on the paternal role in child feeding and indicate the importance of assessing the roles of both parents in shaping the dietary behaviors of Saudi children. In most Arab countries, mothers are typically responsible for preparing meals, whereas fathers are responsible for providing food and grocery. Perhaps, fathers interested in a healthy food environment frequently provide healthy food at home, facilitating their child’s exposure to a HHFE. Future research can be directed toward the role of fathers in implementing feeding practices within Saudi families.

This study has some limitations. Given the bidirectional relationship between maternal and child feeding [17], and due to the nature of our study design, the findings of the present study cannot infer causality. Prospective studies are warranted to investigate the long-term effect of the employed feeding practices on child nutrient intake and diet quality and to identify the direction of the associations. In the present study, dietary recalls were collected using telephone-administered interviews. A previous study which investigated the validity of telephone-administered dietary data suggested that telephone-administered methodology provides acceptable estimates of nutrient intakes [27]. The convenience sampling used to recruit participants may limit the generalizability of our findings. Nevertheless, our study offers new directions to explore the mother–child feeding relationship that may influence a child’s nutrient intake.

## 5. Conclusions

Maternal feeding practices were found to be linked to nutrient intakes in children. Exposing children to greater levels of PE was linked to higher consumption of carbohydrate and dietary fiber and less consumption of fat. Greater exposure to a HHFE was associated with higher consumption of protein and less consumption of free sugar. These findings may direct future studies to incorporate a comprehensive set of feeding practices contextualizing the roles of mothers and fathers. Additional work is also needed to identify factors that may influence the nutrient intake of children and barriers to meeting dietary recommendations. Such research efforts may provide insight into the dynamics of parent–child feeding interaction and guidance for developing nutritional interventions.

## Figures and Tables

**Table 1:** Child characteristics and associations ^a^ with maternal feeding practices.

Child Characteristics	*n* = 424	Pressure to Eat		Healthy Home Food Environment	
		Mean ± SD	*p*-Value	Mean ± SD	*p*-Value
Sex, *n* (%)					
Boys	210 (49.5)	2.83 ± 1.00	0.05	3.36 ± 0.86	0.05
Girls	214 (50.5)	2.65 ± 1.02		3.54 ± 0.81	
Region of residence, *n* (%)					
Western	242 (57.1)	2.73 ± 1.01	0.40	3.41 ± 0.81	0.66
Central	56 (13.2)	2.96 ± 1.05		3.60 ± 0.84	
Eastern	53 (12.5)	2.68 ± 1.00		3.43 ± 0.97	
Southern	48 (11.3)	2.56 ± 0.90		3.51 ± 0.80	
Northern	25 (5.9)	2.76 ± 1.17		3.47 ± 0.91	
Child order among siblings, *n* (%)					
Only child	22 (5.2)	2.91 ± 1.27	0.24	3.15 ± 0.90	0.04 *
Youngest child	128 (30.2)	2.59 ± 1.04		3.35 ± 0.77	
Middle child	135 (31.8)	2.73 ± 0.94		3.55 ± 0.87	
Oldest child	139 (32.8)	2.85 ± 1.00		3.50 ± 0.84	
Maternal education, *n* (%)					
Less than high school	18 (4.2)	2.61 ± 0.88	0.78	3.61 ± 0.85	0.38
High school or diploma	87 (20.5)	2.83 ± 1.06		3.46 ± 0.81	
College degree	270 (63.7)	2.72 ± 1.03		3.41 ± 0.85	
Graduate studies	49 (11.6)	2.73 ± 0.92		3.61 ± 0.82	
Paternal education, *n* (%)					
Less than high school	23 (5.4)	2.60 ± 1.10	0.57	3.35 ± 0.92	0.01 *
High school or diploma	133 (31.4)	2.81 ± 1.11		3.27 ± 0.83	
College degree	205 (48.3)	2.68 ± 0.97		3.53 ± 0.84	
Graduate studies	63 (14.9)	3.62 ± 0.77		3.62 ± 0.77	
Maternal employment status, *n* (%)					
Unemployed	251 (59.2)	2.79 ± 1.03	0.26	3.49 ± 0.83	0.41
Employed	173 (40.8)	2.66 ± 0.99		3.40 ± 0.84	
Father lives with the child, *n* (%)					
Yes	379 (89.4)	2.75 ± 0.99	0.52	3.48 ± 0.85	0.03 *
Sometimes	16 (3.8)	2.67 ± 1.09		3.41 ± 0.69	
No	29 (6.8)	2.59 ± 1.26		3.07 ± 0.70	
Paternal involvement in child feeding, *n* (%)					
Yes	201 (47.4)	2.79 ± 0.99	0.30	3.61 ± 0.84	<0.01 **
Sometimes	125 (29.5)	2.73 ± 0.98		3.38 ± 0.80	
No	98 (23.1)	2.62 ± 1.09		3.22 ± 0.82	
Any breastfeeding during the first six months of life, *n* (%)					
No	55 (13.1)	2.59 ± 1.18	0.22	3.22 ± 0.82	0.02 *
Yes	369 (87.0)	2.76 ± 0.99		3.49 ± 0.84	
Household income in Saudi Riyals, *n* (%)					
<4000	29 (6.80)	2.90 ± 1.06	0.87	3.44 ± 0.93	0.33
4000–6000	62 (14.6)	2.79 ± 0.99		3.36 ± 0.85	
6001–10,000	104 (24.5)	2.75 ± 1.03		3.34 ± 0.81	
10,001–15,000	108 (25.5)	2.67 ± 0.98		3.50 ± 0.86	
>15,000	121 (28.5)	2.71 ± 1.04		3.55 ± 0.80	

^a^ Mann–Whitney and Kruskal–Wallis tests were used. Maternal feeding practices scale scores represent a possible range of 1 to 5. * *p <* 0.05. ** p < 0.01.

**Table 2:** Spearman’s correlation of energy and nutrient intake with maternal feeding practices.

Dietary Intake	Pressure to Eat		Healthy Food Environment	
	r_s_	*p*-Value	r_s_	*p*-Value
Energy (kcal)	0.09	0.07	−0.09	0.06
Carbohydrate (%)	0.12 *	0.02	−0.01	0.85
Protein (%)	−0.07	0.13	0.19 **	<0.01
Fat (%)	−0.17 *	0.03	−0.10 *	0.04
Fiber (g/1000 kcal)	0.13 *	0.01	0.00	0.98
Total sugar (g/1000 kcal)	0.11 *	0.02	−0.13 *	0.01
Free sugar (%)	−0.04	0.40	−0.27 **	<0.01
Calcium (mg/1000 kcal)	0.08	0.11	−0.07	0.16
Iron (mg/1000 kcal)	0.10 *	0.04	−0.04	0.37
Zinc (mg/1000 kcal)	0.08	0.08	0.02	0.63
Vitamin D (ug/1000 kcal)	0.05	0.23	−0.01	0.92
Vitamin C (mg/1000 kcal)	0.11 *	0.02	−0.01	0.80

* *p* < 0.05. ** *p* < 0.01.

**Table 3:** Multiple linear regression analyses of maternal use of pressure to eat on energy and nutrient intakes of children ^a^.

Energy and Nutrient	R^2^	B	Standard Error	95% Confidence Intervals	*p*-Value
Energy (kcal)	0.02	31.9	17.1	−1.74, 65.6	0.06
Carbohydrate (%)	0.04	0.90	0.37	0.19, 1.62	0.01 *
Protein (%)	0.01	−0.31	0.20	−0.70, 0.09	0.12
Fat (%)	0.04	−0.74	0.32	−1.37, −0.11	0.02 *
Fiber (g/1000 kcal)	0.02	1.25	0.47	0.32, 2.18	0.01 *
Total sugar (g/1000 kcal)	0.01	4.59	3.06	−1.42, 10.6	0.13
Free sugar (%)	0.01	−0.67	0.65	−1.94, 0.60	0.30
Calcium (mg/1000 kcal)	0.02	34.0	24.8	−14.7, 82.6	0.17
Iron (mg/1000 kcal)	0.02	0.78	0.40	0.01, 1.56	0.05
Zinc (mg/1000 kcal)	0.02	0.24	0.21	−0.17, 0.65	0.26
Vitamin D (ug/1000 kcal)	0.08	1.73	3.23	−4.63, 8.09	0.59
Vitamin C (mg/1000 kcal)	0.02	6.59	3.54	−0.36, 13.5	0.06

^a^ All models were adjusted for child age and sex, maternal age, and region of residence. * Significant at *p* < 0.05.

**Table 4:** Multiple linear regression analyses of maternal use of a healthy food environment on energy and nutrient intakes of children ^a^.

Energy and Nutrient	R^2^	B	Standard Error	95% Confidence Intervals	*p* Value
Energy (kcal)	0.03	−33.6	20.7	−74.3, 7.23	0.11
Carbohydrate (%)	0.03	−0.25	0.45	−1.13, 0.63	0.58
Protein (%)	0.05	0.99	0.24	0.53, 1.46	<0.01 *
Fat (%)	0.06	−0.74	0.39	−1.51, 0.02	0.06
Fiber (g/1000 kcal)	0.01	−0.43	0.58	−1.56, 0.71	0.46
Total sugar (g/1000 kcal)	0.03	−9.21	3.68	−16.5, −1.97	0.13
Free sugar (%)	0.05	−3.56	0.75	−5.03, −2.09	<0.01 *
Calcium (mg/1000 kcal)	0.03	−24.7	30.0	−83.7, 34.3	0.41
Iron (mg/1000 kcal)	0.02	−0.31	0.48	−1.25, 0.64	0.52
Zinc (mg/1000 kcal)	0.02	0.02	0.25	−0.48, 0.52	0.08
Vitamin D (ug/1000 kcal)	0.09	−1.50	3.92	−9.20, 6.20	0.70
Vitamin C (mg/1000 kcal)	0.02	−0.21	4.30	−8.66, 8.25	0.96

^a^ All models were adjusted for child age and sex, region of residence, order of child, paternal education, paternal involvement in feeding, paternal residency status with child, and breastfeeding status during the first six months of the child’s life. * Significant at *p* < 0.05.

## Data Availability

The data used to support the findings of this study are available from the corresponding author upon request.
